# Migrants in Psychiatric Care: A Retrospective Study from a Psychiatric Unit in Tunisia

**DOI:** 10.1192/j.eurpsy.2025.401

**Published:** 2025-08-26

**Authors:** D. Mezri, A. Gharbia, S. Hamzaoui, U. Ouali, A. Aissa, R. Jomli

**Affiliations:** 1 Avicenne, Razi hospital, Mannouba, Tunisia

## Abstract

**Introduction:**

Migration, whether legal or illegal, is a growing phenomenon in Tunisia and can bring significant mental health challenges. Migrants often experience a decompensation of pre-existing psychiatric disorders, the development of new mental health issues, or travel driven by a delusion, known as “pathological travel.” These concerns emphasize the need for specialized psychiatric and social care for this vulnerable group, who endure considerable stress throughout their migration. However the quality of care can also depend on the support from their home countries, where stigma and identification issues with consulates can limit their access to help.

**Objectives:**

To study the different psychiatric pathologies observed among this population and to determine the number of cases of pathological travel.

**Methods:**

It’s a retrospective study. We reviewed the files of all patients who were hospitalized in the Avicenne Psychiatric Department of Razi Hospital between January 2022 and December 2023.

**Results:**

We identified 19 patients and found 17 files. There were 11 men and 6 women, with an average age of 33 years (ranging from 20 to 54 years). The majority have a university-level education (52%) and with a history of psychiatric illness (58%). In total, 41.2% were from the Maghreb, 41.2% from Africa, 11.7% from Europe, and 5.9% from the Americas. The causes of migration to Tunisia were, for economic reasons (29%), for studies (11%), for seeking treatment for a pre-existing psychiatric condition (11%), marriage to a Tunisian partner (11%) and as part of a pathological travel (34%). In 35% of cases, the migration was clandestine and illegal. The reason for hospitalization was behavioral disorder in 64.8%, incoherent speech in 29.4% and suicide attempt in 5.8%. Among our patients, 28% have bipolar disorder, 17% have schizophrenia, 11% have brief psychotic disorder, 5% have depression, 5% have schizoaffective disorder, 5% have delusional disorder, and 5% alcohol use disorder. The diagnoses for the rest of patients were unspecified. In terms of social support we were able to contact the families in 62% of the cases. We succeeded in getting a response from the consulate of the native country in 17% and we collaborated with an International Organization in 5%.

**Conclusions:**

Our study shows the complex psychiatric needs of migrants in Tunisia, with a range of mental health disorders, including cases of pathological travel. Economic reasons and clandestine migration were common factors. Despite efforts, social support remains limited, with minimal consular and organizational collaboration, economic challenges, emphasizing the need for stronger international and social support systems.

**Disclosure of Interest:**

None Declared

